# Effect of Ether and Chloroform on Animal Temperature

**Published:** 1848

**Authors:** 


### Article XI.—*Effect of Ether and Chloroform on Animal Temperature.*

MM. Dumeril and Demaquay have communicated to the Academy of Sciences a series of experimental researches on the modification of animal temperature produced by ether and chloroform, and on the physiological action of those agents.

They state that the temperature is peculiarly lowered in animals submitted to the influence of the vapor of those intoxicating agents: that this depression is greater from ether. This effect is constant, whether the vapor be introduced into the respiratory passages, or into the rectum. Section of the pneumogastric nerves, almost simultaneously with the application of the inhaler to the mouth, does not modify the results obtained when those nerves are uninjured. The temperature is depressed also, during reaction, consequent on the section of one of the pneumogastric nerves, twenty-four or forty-eight hours before inhalation. The authors further believe that these facts warrant the conclusions, that ether does not act primarily in the manner of an asphyxiating agent, but that the asphyxia induced is but a secondary effect following the penetration of its vapor into the economy; that the phenomena of etherization set out from the disorder they induce in the central nervous system; that the asphyxia is but consecutive, and if fatal, it is because etherization has lasted so long as to abolish the functions of the medulla oblongata, the last part of the nervous centres acted upon by the agent.

They further state that a loss of sensation, together with a depression of temperature, is brought also by brandy; but that narcotics, instead of lowering animal heat, raise it, save for a very brief period, immediately after their ingestion.

The injection of ether-vapor into the rectum, shows that apart from the disorder of the respiratory function, there is a depression of temperature, which must arise from a special action of the nervous system. If, then, the source of animal heat be in the process of blood-making, and the latter be immediately dependent on the nervous system, the possibility of a modification of temperature by any cause acting primarily upon that system is at once seen.

As a further result of their experiments, MM. Dumeril and Demarquay state that the action of ether and of chloroform is rapidly fatal, since they have seen it destroy dogs in thirty-five or forty-five minutes, and even in less time, with reference to chloroform.—*London Lancet.*

